# Effects of Process Parameters on the Mechanical Properties and Microstructure of Additively Manufactured Carbon Black Particles-Reinforced Thermoplastic Polyurethane Composite Samples

**DOI:** 10.3390/polym17030426

**Published:** 2025-02-06

**Authors:** Fatima Hira, Muhammad Asif, Hammad Ullah, Imran Khan, Ghulam Hussain, Muhammad Amir, Mohammed Alkahtani

**Affiliations:** 1 Department of Physics, Shaheed Benazir Bhutto Women University, Peshawar 25000, Pakistan; 2 Department of Mathematics, University of Peshawar, Peshawar 25120, Pakistan; 3 Department of Computer Science, Sarhad University of Science & Information Technology (SUIT), Peshawar 25000, Pakistan; 4 Department of Mechanical Engineering, University of Engineering and Technology Peshawar, Peshawar 25120, Pakistan; 5 Mechanical Engineering Department, College of Engineering, University of Bahrain, Isa Town 32038, Bahrain; ghussain@uob.edu.bh; 6 Department of Data Science, Friedrich-Alexander Universität Erlangen-Nürnberg, 91054 Erlangen, Germany; 7 Department of Industrial Engineering, College of Engineering, King Saud University, Riyadh 12372, Saudi Arabia; moalkahtani@ksu.edu.sa

**Keywords:** 3D printing, fused filament fabrication, parametric study, optimization, mechanical properties, ANOVA

## Abstract

Additive manufacturing (AM) techniques make fabricating complex designs, prototypes, and end-user products possible. Conductive polymer composites find applications in flexible electronics, sensor fabrication, and electrical circuits. In this study, thermoplastic polyurethane (TPU)-based conductive polymer composite samples were fabricated via fused filament fabrication (FFF). The effects of three important process parameters, including infill density (ID), layer thickness (LT), and fan speed (FS), on various mechanical properties (tensile and compressive properties) were investigated. It was observed that all the considered process parameters affect the mechanical properties, and they are significant parameters, as per the analysis of variance (ANOVA). From scanning electron microscopy (SEM) and optical microscopy, various combinations of parameters such as low ID, high LT, and high FS resulted in the formation of defects such as voids, cracks, and warping, which resulted in low mechanical properties. Finally, process parameter optimization was performed, resulting in a conductive polymer composite with the best possible combination of mechanical properties at high ID, low LT, and medium FS.

## 1. Introduction

Additive manufacturing (AM) is also known as 3D printing (3DP). AM techniques add a layer-by-layer deposition of material for creating three-dimensional (3D) objects and prototypes [1,2]. This technology is used in various applications like aerospace, biomedical, automotive industries, and sensor fabrication [3,4]. Raja et al. [5] highlighted the importance of AM in aerospace industries because of its ability to fabricate complex and high-performance products easily without tooling. As per the American Society for Testing and Materials (ASTM) International, AM processes include selective laser sintering (SLS), stereolithography (SLA), extrusion-based AM such as fused filament fabrication (FFF), laminated object manufacturing (LOM), and electron beam manufacturing (EBM) [6]. FFF is the most common technique, where the thermoplastic material is melted and extruded layer by layer, which, on solidification, forms the desired 3D structure [7]. In FFF, there are various process parameters like ID, LT, raster angle (RA), nozzle temperature (NT), bed temperature (BT), printing speed (PS), and others, which affect the strength and accuracy of the fabricated components [8,9]. F. Finollosa [7] reported using the FFF technique in the medical sector to create surgical training prototypes that are used during surgery.

Many thermoplastic materials in filaments can be fed into the extruder by setting the nozzle at the desired temperature for fabricating FFF products [10]. Arup Dey [11] reported that different thermoplastic materials have different properties, but in the FFF technique, filament selection generally depends upon their applications. He further listed the most widely used filaments including acrylonitrile butadiene styrene (ABS), thermoplastic polyurethane (TPU), polylactic acid (PLA), polycarbonate (PC), nylon, and many others. The recent trend in using extrusion-based AM is to produce functional devices and products using composite filaments [12]. Composites are a class of materials consisting of two different materials that are physically combined, not chemically, and they can be used in specific applications where the individual constituent materials cannot perform well [13]. Composite-based products can be used in various applications, such as aerospace, automotive, biomedical, and sports industries [14,15] due to their enhanced functional and mechanical properties. Polymeric composites can be of three types: (1) particle-reinforced, (2) fiber-reinforced, and (3) laminated or sheet composites [16,17]. There have been studies on producing all three types of composites via AM processes [14,18,19,20]. Some composite filaments have been produced by recycling polymer materials such as polypropylene and reinforcing them with particles or fibers such as carbon fibers or basal salt, etc. It also contributes to the innovations in the circular economy of AM [21,22,23].

There are studies on the fabrication of FFF-based products utilizing multi-material-based 3DP for functional parts, such as the fabrication of sensors. Kim et al. [24] utilized the FFF technique to produce multiaxial force sensors. In his work, the structural component was fabricated using TPU filaments, while the sensing part was printed from carbon nanotubes (CNT) reinforced-TPU composite filaments. Rasheed et al. [25] investigated the production and mechanical properties of an alternate-layered bi-material 3D printed laminated composite made via a multi-materials-based 3DP technique. The effects of four printing parameters, ID, PS, the number of layers, and BT, were investigated in terms of the mechanical properties and printing time. It was observed that an increase in PS resulted in decreasing strength, and the converse was true for the number of layers. It was further reported that the optimum parameters for achieving maximum tensile strength included 75% ID, PS = 20 mm/s, 30 layers per part, and a BT of 100 °C. Similarly, Qingxi Hu [26] utilized the FFF technique to print continuous carbon fiber specimens and investigate the mechanical properties of the resulting composite. Three printing parameters were varied, including NT, PS, and LT. After experimentations, the optimum parameters for maximum flexural strength were NT = 230, PS = 60, and LT = 0.6.

TPU is a commonly used FFF polymeric material. It is known for its stretchability, which opens up possibilities in smart wearables, such as smart clothing, and is widely used in several applications, including automotive, aerospace, biomedical, and sports industries [27,28]. Adding conductive particles such as graphite, carbon black, or metallic particles into TPU material can increase the conductivity of TPU, which can then be used for various applications. For better mixing of reinforcing particles in the polymeric matrix for filament production, primarily, a twin screw extruder is utilized [29,30]. Conductive TPU-based filaments have been used in a few studies for the fabrication of multiple sensing structures and electrodes [31,32,33]. Riley et al. [34] utilized the FFF technique to print mechanical sensors, known as mechanosensors, which consisted of a dome with a piezo-resistive strip at the base. The carbon black-reinforced Recreus Conductive Filaflex material was employed for the piezo-resistive strip, and the dome and base were composed of non-conductive TPU material. The material’s signal amplification, signal filtering, and time-dependent behavior were investigated in that study. It was concluded that the TPU-based conductive material shows significant viscoelastic behavior, and the magnitude of external forces was successfully stored in the material. Moreover, Z. Aloqalaa [35] summarized various types of conductive filaments for use in the FFF technique.

Mechanical properties are highly important in engineering applications because they predict materials’ behavior and response under external loads [36]. Numerous studies have been performed on the effects of AM process parameters on mechanical properties, such as tensile yield strength (TYS), ultimate tensile strength (UTS), flexural strength, Young’s modulus (E), etc. [37,38]. These mechanical properties of the 3D-printed parts depend on the 3DP process parameters of PS, NT, BT, ID, infill pattern (IP), LT, RA, and many more [39]. Antonio et al. [40] performed a parametric study of PLA-based parts fabricated via the FFF method. It was observed that a decrease in RA increased the tensile strength. However, with an increasing number of shells, the tensile strength increased slightly, followed by a sharp non-linear decrease. The effects of LT and the number of shells were also discussed in the case of Young’s modulus.

Conductive TPU-based filaments, such as conductive Filaflex, are a new type of material. They have many potential applications in numerous sectors, such as sensor fabrication, wearable electronics, and biomedical industries. In very few studies, these types of materials have been employed mostly for sensing elements’ fabrication and feasibility studies [31,32,33]. According to the authors’ knowledge, there is a lack of literature and studies on the mechanical performance of conductive TPU-based materials. The main objective of this research work was to fill this research gap so that the research community also knows about the mechanical performance of these materials. This study investigated the effects of 3DP process parameters on the mechanical properties of conductive TPU-based material. The effects of LT, IF, and fan speed (FS) were investigated on the mechanical properties such as TYS, UTS, failure strain (F-STRAIN), E, compressive modulus (CM), and compressive strength at 10% strain (CS10). ANOVA analysis was conducted to determine the significance of each parameter, followed by microscopy of the samples. Finally, the optimum parameters for fabricating electrically conductive TPU-based materials via FFF were proposed.

## 2. Materials and Methods

This study utilized Conductive Filaflex (Recreus, Alicante, Spain) filaments with a diameter of 1.75 mm. Conductive Filaflex is a flexible, electrically conductive TPU-carbon black filament with 92 A Shore hardness. According to the manufacturer, it is perfect for fabricating electrically conductive circuits and ideal for manufacturing wearable and medical devices like ECG patches, electronic circuits, etc. This filament has an electrical resistance of approximately 3.9 Ω-cm [41].

In this study, the effect of three 3DP process parameters on the mechanical properties was investigated. These parameters included LT, ID, and FS with varying combinations of the levels of the parameters, as shown in Table 1. The values of the levels of parameters were selected on the basis of preliminary experimentation and testing of the filament. A response surface method (RSM) design was utilized to formulate an experimental plan using statistical software. This plan consisted of 13 experiments, as shown in Table 2. Other process parameters were kept constant in all the experiments of the DOE plan, and their values were selected as per the preliminary experimentation, the manufacturer’s recommendations, and the literature. These constant parameters and their values included a cubic subdivision infill pattern, a nozzle temperature of 250 °C, a bed temperature of 60 °C, and a nozzle diameter of 0.4 mm. Similarly, a horizontal build orientation, a top/bottom thickness of 0.84 mm, a retraction speed of 40 mm/s, a print flow of 100%, and a retraction distance of 3 mm were also kept as constant parameters. Tensile yield strength (TYS), ultimate tensile strength (UTS), modulus of elasticity (E), failure strain (F-STRAIN), compressive modulus (CM), and compressive strength at 10% strain (CS10) were considered as the output responses of this study.

SolidWorks software (student version 2020) was utilized for CAD designs of dog-bone specimens according to the ASTM D638 Type 1 standard [42] with a thickness of 5 mm, a gauge length of 50 mm, and a grip distance of approximately 30 mm. Moreover, the compressive tests were conducted according to the ASTM-D695 compressive test standard [43], with a square cross-section of 12.7 mm on each side and a height of 25.4 mm. The STL files were also generated by SolidWorks software, and the CURA slicer program was used for parametric settings. Three samples for each experiment were printed using the Creality CR-10 3D printer, and an average of the three values of the output properties are reported in this paper. Creality, officially known as Shenzhen Creality 3D Technology Co., Ltd. (Shenzhen, China), is a Chinese 3D printer manufacturing company established in 2014. The Creality CR-10 3D printer has a build volume of 300 × 300 × 400 mm, a maximum print speed of 150 mm/s, a 0.4 mm nozzle diameter, and a nozzle and bed temperature of 260 °C and 100 °C, respectively. Numerous filaments, including PLA, PETG, TPU, PET, and ABS, show printing compatibility for producing 3D models with this printer.

**Figure 1:**
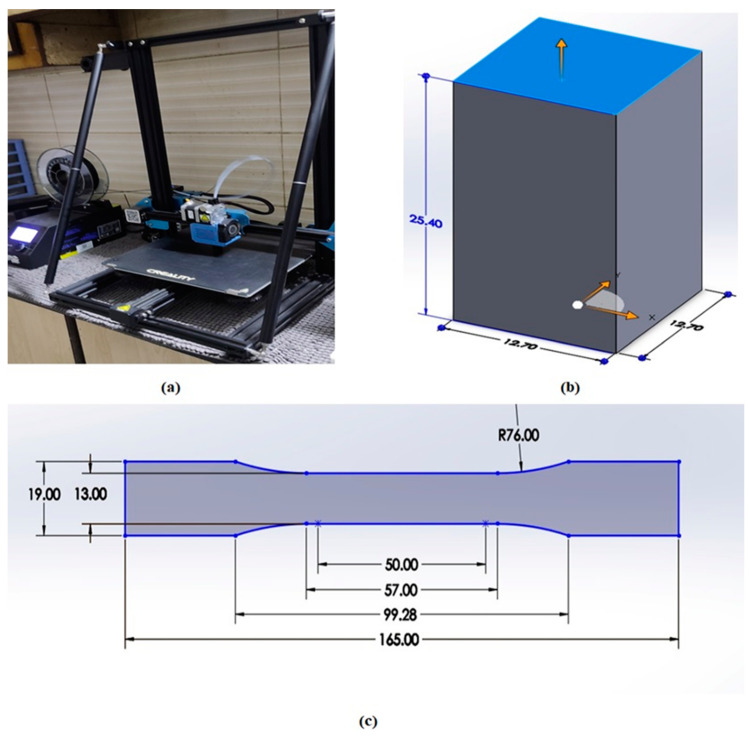
(**a**) Creality CR10 3D printer. (**b**) ASTM-D695 compressive test sample (dimensions in mm). (**c**) ASTM-D638 tensile test sample (dimensions in mm).

Tensile and compressive testing of the specimens was carried out on the SHIMADZU Autograph AGS-X Series Precision Universal Tester (50 KN). The speed of 2 mm/min was maintained throughout the testing. Optical microscopy was performed using a LEICA EC3 microscope with a high resolution of about 3.1 megapixels and a high-speed digital color camera that offers real-time imaging at 20 FPS. The LAS EZ application suite integrates a microscope and digital camera into one common imaging system. The outcome is precise, sharp images that can be saved, displayed in an integrated gallery, and reviewed at any time. According to ASTM standards, Figure 1 shows the dimensions of the 3D printer, tensile sample, and compressive sample. Printing and testing of the test samples were performed at room temperature (25 ± 3 °C), atmospheric pressure (101 ± 4 KPa), and a relative humidity of 50 ± 3%.

## 3. Results and Discussion

### 3.1. Mechanical Testing Results

Table 3 shows the mechanical properties of the printed samples after tensile testing. It can be observed that Experiment # 3 (LT = 0.12, ID = 85, and FS = 60) showed the highest UTS, TYS, CM, and CS10. This might be due to the high ID and low LT levels, as a high ID means the material or sample is relatively solid with slight hollowness on the inside, and low LT might have resulted in better adhesion between the layers. On the other hand, Experiment # 2 (LT = 0.28, ID = 50, and FS = 60) showed the lowest TYS, UTS, CM, and CS10. This might be due to the low ID and high LT levels, as low ID means the material or sample is mostly hollow on the inside, and high LT might have resulted in inferior adhesion between the layers. Similarly, Experiment # 12 (LT = 0.2, ID = 85, and FS = 100) and Experiment # 4 (LT = 0.28, ID = 85, and FS = 60) showed the maximum E, possibly due to larger LT and FS values resulting in higher cooling rates. On the other hand, Experiment # 10 (LT = 0.2, ID = 85, and FS = 20) showed the highest F-STRAIN, possibly due to lower cooling rates. Moreover, Experiment # 5 showed the minimum E, and Experiment # 11 showed the lowest F-STRAIN. It can be inferred from Table 3 that no single experiment exhibited the highest or lowest of all mechanical properties.

### 3.2. Microscopic and Visual Analysis

**Figure 2:**
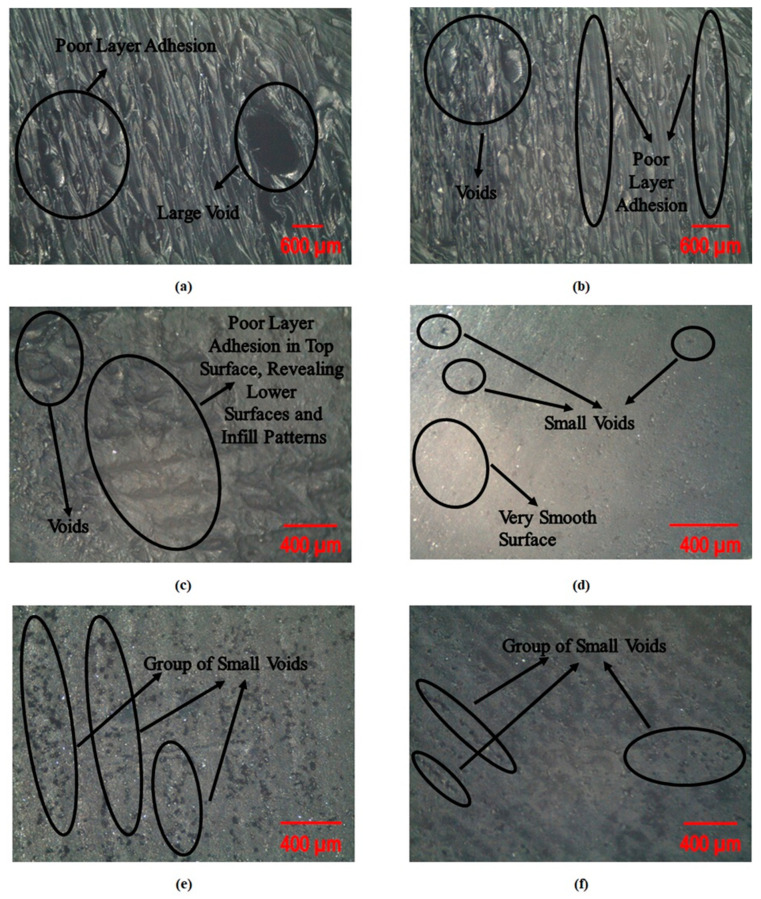
SEM micrographs of selected experiments: (**a**,**b**) Experiment # 2; (**c**) Experiment # 11; (**d**) Experiment # 3; (**e**,**f**) Experiment # 13.

Figure 2 shows the SEM micrographs of selected samples. It can be observed from Figure 2a–c that the process parameters of Experiments # 2 and 11 resulted in poor layer adhesion and voids, which resulted in lower mechanical properties such as TYS, UTS, CM, and CS10. This can be attributed to the lower ID and high LT, as already discussed in the literature [16]. Similarly, in Figure 2d, Experiment # 3’s process parameters resulted in an overall smooth surface with tiny voids at some places, which resulted in better mechanical properties. This might be due to high ID and low LT, which resulted in better layer adhesion and, thus, better resistance to deformation. Moreover, in Figure 2e,f, it can be observed that Experiment # 13 resulted in groups of smaller voids and small cracks, which resulted in intermediate mechanical properties due to the medium ID and LT.

**Figure 3:**
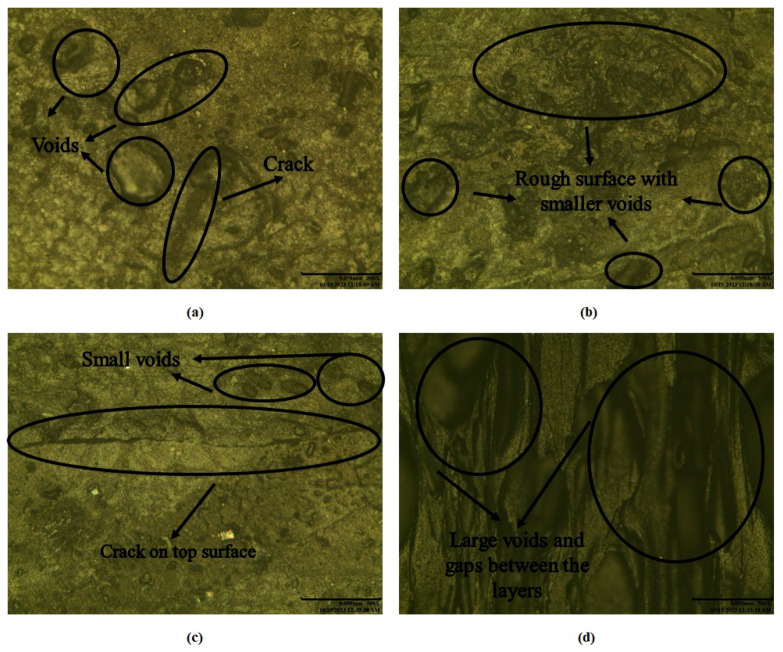
Optical microscopic images of compressive samples: (**a**) top view of Experiment # 9, (**b**) top view of Experiment # 6, (**c**) Top view of Experiment # 2, and (**d**) side view of Experiment # 2.

Figure 3 shows the optical microscopic images of selected compressive samples. It can be observed that lower ID resulted in significant voids and rough surfaces in Experiments # 2 and 9, resulting in lower compressive properties, as evident in Figure 3a,c,d. Similarly, Experiment # 6 resulted in medium compressive properties, mainly because of the medium ID level, resulting in relatively smaller voids in compressive samples.

**Figure 4:**
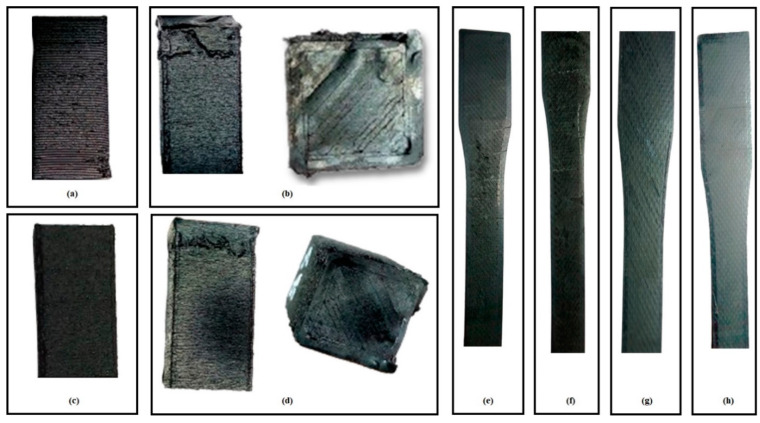
High-resolution camera images of compressive and tensile samples: (**a**) compressive Experiment # 13, (**b**) compressive Experiment # 2, (**c**) compressive Experiment # 3, (**d**) compressive Experiment # 9, (**e**) tensile Experiment # 2, (**f**) tensile Experiment # 9, (**g**) tensile Experiment # 11, and (**h**) tensile Experiment # 3.

Moreover, Figure 4 shows the naked eye defects and surface nature before mechanical testing. It can be observed that Experiment # 2 (Figure 4b) and Experiment # 9 (Figure 4d) resulted in localized misaligned layers, poor layer adhesion, and defective top and side surfaces. Similarly, Figure 4a shows that Experiment # 6 also showed localized defects at the right bottom corner and some voids (which can be seen with the naked eye). However, Experiment # 3 (Figure 4c) resulted in a fine, smooth surface and good layer adhesion. Moreover, Figure 4e,f shows defects (even with the naked eye) in the tensile samples of Experiments # 2 and 9. However, Experiments # 3 and 11 showed a better surface finish (Figure 4g,h) and few defects, resulting in better tensile properties.

**Figure 5:**
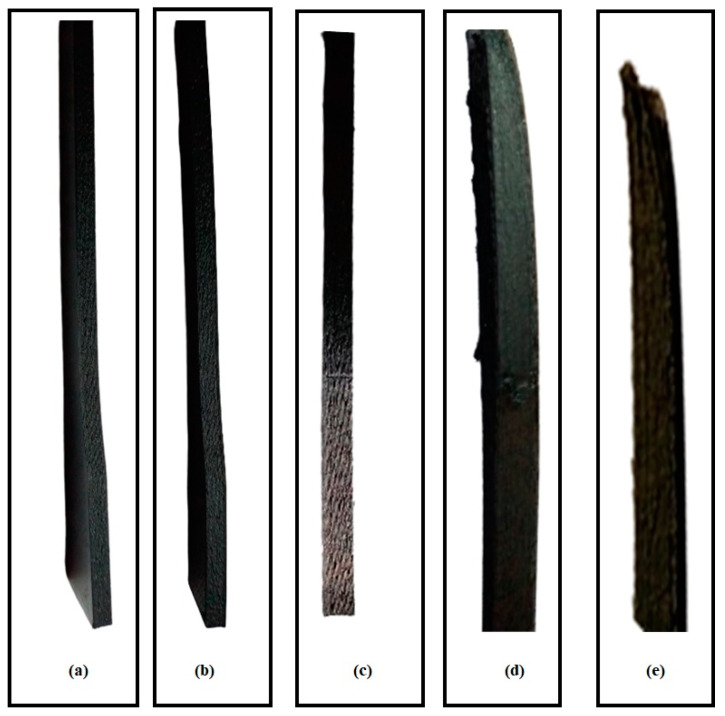
Warping effects in (**a**) Experiment # 3 (no warping), (**b**) Experiment # 5 (no warping), (**c**) Experiment # 10 (no warping), (**d**) Experiment # 7 (warping at the upper end), and (**e**) Experiment # 8 (warping at the upper end).

In addition, Figure 5 shows the warping effects in various experiments. It can be observed that Experiments # 3, 5, and 10 did not exhibit warping effects, as evident from Figure 5a–c. However, Experiments # 7 and 8 exhibited warping effects, as shown in Figure 5d,e. This is mainly due to higher FS, which rapidly cools down the extruded layer, especially the first few layers. Therefore, the layers exhibited poor adhesion to the print bed after some layers had been laid up (printed).

### 3.3. ANOVA Analysis

ANOVA analysis was conducted to determine the significant parameters that affect the output responses, as shown in Table 4. Regression analysis was performed using the stepwise method at an alpha value of 0.15. For this purpose, insignificant model terms were removed, and relevant terms within the regression model could be recognized. It can be observed from the ANOVA table that in the case of TYS, the LT, and ID were significant stand-alone parameters with a *p*-value less than 0.05, while FS did not significantly affect the TYS. In the case of UTS, the LT, ID, FS, and (FS*FS) were significant parameters. However, for F-STRAIN, LT, ID, FS, and (ID*ID) were significant terms with a *p*-value less than 0.05. Similarly, for TM, the significant process parameters included LT, ID, FS, (FS*FS), (LT*ID), and (ID*FS). The regression equations after ANOVA analysis (via statistical software) are below. The R^2^ value of all the regression models was more than 90%, showing the reliability and adequacy of the models.

TYS=−86−1353 LT+18.60 ID

UTS=163−2401 LT+52.98 ID+10.42 FS−0.1129 FS∗FS

E=−1.37+39.06 LT+0.1986 ID+0.2469 FS−0.367 LT∗FS−0.001607 ID∗FS

F−STRAIN=135.6−53.7 LT−2.28 ID−0.1256 FS+0.02416 ID∗ID

CM=−4.59+8.08 LT+0.5161 ID−0.436 LT∗ID

CS10=5518.6−1114.1 LT−142.68 ID+1.014 FS+1.35347 ID∗ID−0.00821 ID∗FS

### 3.4. Effects of Process Parameters on Mechanical Properties

**Figure 6:**
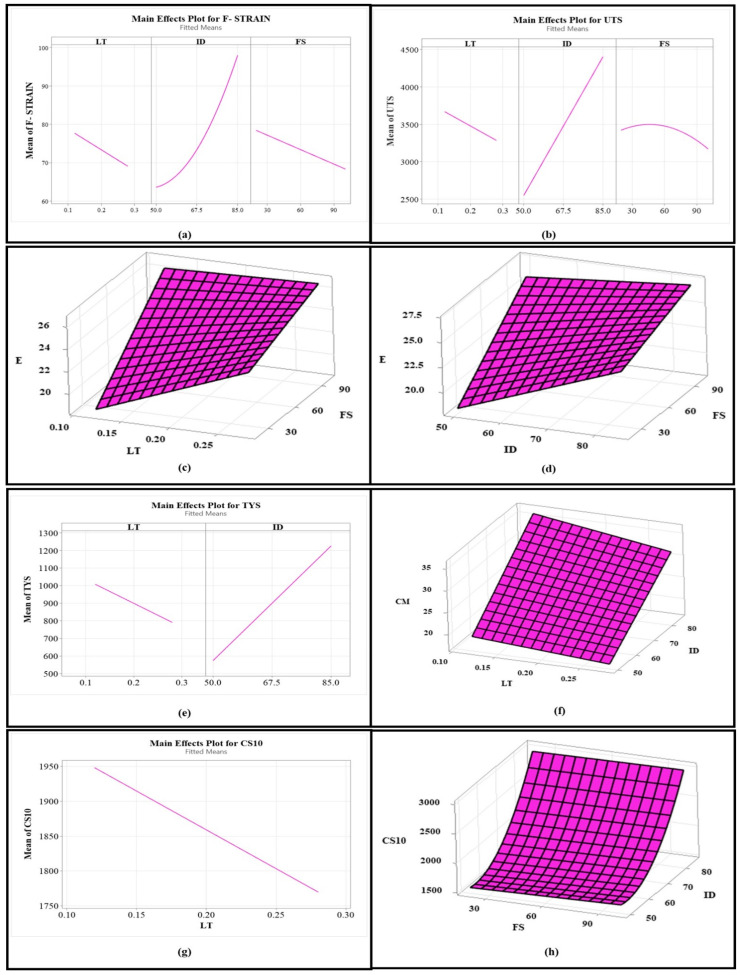
Effects of process parameters on the mechanical properties: (**a**) main effects plot for F-STRAIN (effects of LT, ID, and FS), (**b**) main effects plot for UTS (effects of LT, ID, and FS), (**c**) interaction surface plot of LT*FS for TM, (**d**) interaction surface plot of ID*FS for TM, (**e**) main effects plot for TYS (effects of LT and ID), (**f**) interaction surface plot of ID*LT for CM, (**g**) main effects plot for CS10 (effects of LT), and (**h**) interaction surface plot of ID*FS for CS10.

Figure 6 shows the main effects plots and interaction plots (surface plots), showing the effects of significant process parameters on various mechanical properties. It can be observed from Figure 6a that F-STRAIN increased non-linearly with an increase in ID, and it also increased with a decrease in LT and FS. Moreover, UTS slightly increased and then abruptly decreased with an increase in FS, as shown in Figure 6b. However, an increase in ID and a decrease in LT resulted in a linear increase in UTS. The same effect is true for TYS, as shown in Figure 6e. Similarly, in the case of TM, an increase in FS increased E, as shown in Figure 6c,d. In addition, an increase in LT and ID resulted in a significant increase in E at low FS and little increase in E at a high FS, as shown in Figure 6c,d. Similarly, an increase in ID at any value of LT increased CM, and an increase in LT at any value of ID resulted in a decrease in CM, as observable from Figure 6f. Lastly, CS10 decreased as LT increased (Figure 6g). However, CS10 slightly increased with an increase in FS at a low ID, and CS10 abruptly increased as ID increased, as shown in Figure 6h.

### 3.5. Optimization

While opting for composite filaments for applications, mechanical properties with favorable results should be considered. In this case, maximizing mechanical properties such as F-STRAIN, E, UTS, TYS, CM, and CS10 is desired. High F-STRAIN ensures flexibility and halts abrupt failure, high E upholds structural integrity and the configuration’s reliability, and high UTS results in enduring intense loads without fracturing. Similarly, high TYS avoids permanent deformation, and so does CS10. However, industrial applications need a balance in their mechanical properties for near-ideal performance, as one combination of process parameters may not offer the highest of all desirable properties. Therefore, statistical software has suggested a solution for the optimum parameters considering an LT of 0.12 mm, an ID of 85%, and an FS of 60 % with a desirability of 0.8881, which is closer to 1. These optimum parameters are anticipated in a near-ideal combination of a maximum F-STRAIN of 102.25%, a maximum E of 24.17 MPa, a UTS of 4596.7 KPa, a maximum TYS of 1333.25 KPa, a maximum CM of 35.81 MPa, and a maximum CS10 of 3054.62 KPa. For validation, samples were printed again, and the mechanical properties were measured experimentally, closer to the predicted optimum properties (within a 5% error range). Experimentally, at the suggested optimum process parameters, the electrically conductive composite resulted in 107.1% (F-STRAIN), 24.71 MPa (TM), 4639.7 KPa (UTS), 1409.25 KPa (TYS), 36.23 MPa (CM), and 3061.1 KPa (CS10). Table 5 shows the optimization results of the statistical software.

## 4. Conclusions

In this study, the fabrication of conductive polymer composite samples was carried out successfully via the FFF technique. The effects of process parameters on mechanical properties such as tensile strength, Young’s modulus, strain, compressive strength, and modulus were investigated. ANOVA analysis was also conducted to determine the significant parameters for each output property. It can be concluded that the FFF process parameters considered in this study significantly affected the mechanical properties of the fabricated composite. Moreover, these parameters also affected the microstructure and defect formation. Defects formed at low ID, high LT, and high FS. High ID with low LT and medium FS has been suggested as the optimum FFF parameters for the fabrication of conductive Filaflex-based composites, which resulted in an optimum combination of mechanical properties. The conductive TPU-based composite finds applications in flexible electronics, electrical circuits, etc. Future studies can also investigate the effects of other 3DP process parameters on the electrical and mechanical properties of the same materials and use them to fabricate sensors or other devices.

## Figures and Tables

**Table 1:** Levels of ID, FS, and LT utilized in the parametric study.

Parameter	Short Form	Unit	Level 1	Level 2	Level 3
Infill density	ID	%	50	67.5	85
Fan speed	FS	%	20	60	100
Layer thickness	LT	mm	0.12	0.2	0.28

**Table 2:** The experimental plan of this study.

EXP. #	LT	ID	FS	EXP. #	LT	ID	FS
1	0.12	50	60	8	0.28	67.5	100
2	0.28	50	60	9	0.2	50	20
3	0.12	85	60	10	0.2	85	20
4	0.28	85	60	11	0.2	50	100
5	0.12	67.5	20	12	0.2	85	100
6	0.28	67.5	20	13	0.2	67.5	60
7	0.12	67.5	100	-			

**Table 3:** Mechanical test results.

EXP. #	TYS (KPa)	UTS (KPa)	E (MPa)	F-STRAIN (%)	CM (MPa)	CS10 (KPa)
1	691.1	2634.2	19.2	67.9	19.41	1669
2	446.2	2337.5	22.9	58.7	17.11	1493
3	1437.9	4687.6	24.8	105.6	36.12	3058
4	1156.3	4259.4	26.7	86.7	31.38	2871
5	934.4	3616.8	18.7	75.3	27.87	1929
6	745.1	3274.7	23.7	73.5	23.99	1758
7	927.8	3367.8	26.1	73.9	27.58	1964
8	777.7	2898.0	26.4	69.4	24.85	1785
9	598.0	2529.7	18.9	70.9	18.47	1555
10	1163.7	4267.2	23.6	110.2	33.21	2951
11	637.0	2358.0	26.5	57.1	18.69	1609
12	1219.1	4062.3	26.7	89.3	33.03	2982
13	958.2	3468.7	24.5	74.9	26.09	1859

**Table 4:** ANOVA analysis of TYS, UTS, F-STRAIN, TM, CM, and CS10.

Source	TYS		UTS		F-STRAIN		E		CM		CS10	
	F-Value	*p*-Value	F-Value	*p*-Value	F-Value	*p*-Value	F-Value	*p*-Value	F-Value	*p*-Value	F-Value	*p*-Value
Model	121.22	0	276.51	0	26.08	0	0	32.16	1368.52	0	44,738.73	0
LT	24.13	0.001	44.13	0	5.37	0.049	22.72	0.002	201.02	0	3211.72	0
ID	218.32	0	1028.1	0	85.49	0	39.11	0	3891.69	0	193,620.1	0
FS	-	-	18.78	0.003	7.34	0.027	82.75	0	-	-	136.52	0
FS*FS	-	-	15.01	0.005	-	-	-	-	-	-	-	-
LT*FS	-	-	-	-	-	-	8.45	0.023	-	-	-	-
LT*ID	-	-	-	-	-	-	-	-	12.85	0.006	-	-
ID*FS	-	-	-	-	-	-	7.75	0.027	-	-	6.68	0.036
ID*ID	-	-	-	-	6.12	0.038	-	-	-	-	26,718.62	0

**Table 5:** Optimum parameters suggested by statistical software, along with the predicted output responses values.

Input Process Parameters			Predicted Output Responses							Composite Desirability
LT	ID	FS	CM Fit	CS10 Fit	F-Strain Fit	UTS Fit	MoT Fit	YS Fit	E Fit	
0.12	85	60	35.80	3054.62	102.25	4596.7	489.46	1333.24	24.1712	0.8881

## Data Availability

The original contributions presented in this study are included in the article. Further inquiries can be directed to the corresponding author(s).
